# Metabolic response to CNS infection with flaviviruses

**DOI:** 10.1186/s12974-023-02898-4

**Published:** 2023-09-29

**Authors:** Marta Dobrzyńska, Anna Moniuszko-Malinowska, Elżbieta Skrzydlewska

**Affiliations:** 1 Department of Analytical Chemistry, Medical University of Białystok, Białystok, Poland; 2 Department of Infectious Diseases and Neuroinfections, Medical University of Bialystok, Zurawia 14, 15-540 Bialystok, Poland

**Keywords:** Flaviviruses, Viral infections, CNS, Neuroinfections, Oxidative stress, Inflammation

## Abstract

Flaviviruses are arthropod-borne RNA viruses found worldwide that, when introduced into the human body, cause diseases, including neuroinfections, that can lead to serious metabolic consequences and even death. Some of the diseases caused by flaviviruses occur continuously in certain regions, while others occur intermittently or sporadically, causing epidemics. Some of the most common flaviviruses are West Nile virus, dengue virus, tick-borne encephalitis virus, Zika virus and Japanese encephalitis virus. Since all the above-mentioned viruses are capable of penetrating the blood–brain barrier through different mechanisms, their actions also affect the central nervous system (CNS). Like other viruses, flaviviruses, after entering the human body, contribute to redox imbalance and, consequently, to oxidative stress, which promotes inflammation in skin cells, in the blood and in CNS. This review focuses on discussing the effects of oxidative stress and inflammation resulting from pathogen invasion on the metabolic antiviral response of the host, and the ability of viruses to evade the consequences of metabolic changes or exploit them for increased replication and further progression of infection, which affects the development of sequelae and difficulties in therapy.

## Background

The twenty-first century is characterized by intensifying climate change and human activities that disrupt the entirety of the ecosystem, causing the spread of zoonotic microorganisms, which also affect the human population. In addition, the growing demand for food of animal origin has led to an increase in the number of livestock, creating more opportunities for pathogens to interbreed. Each year, zoonotic diseases cause more than 1 billion human infections, including more than 1 million deaths [1]. Among other factors, this contributes to the emergence of new and the recurrence of seemingly forgotten diseases. This especially concerns infectious diseases.

The above reasons favor the emergence of diseases in new regions (e.g., malaria, Chikungunya fever) and the resurgence of others, such as tuberculosis [2]. It should be noted that many pathogens that are dangerous to humans come from animals. An extreme example is malaria—already a huge threat as the world’s most widespread infectious disease, with nearly 247 million cases a year—which is also affected by the warming climate that favors the spread of malaria-carrying mosquitoes [https://www.who.int/news-room/fact-sheets/detail/malaria]. Besides mosquitoes, ticks are one of the most important vectors of infectious diseases, also affected by climate change. Therefore, tick-borne diseases also spread into new territories. Both mosquitoes and ticks carry a variety of pathogens, i.e., viruses, bacteria, and protozoa, which are dangerous both to humans and animals.

**Fig. 1:**
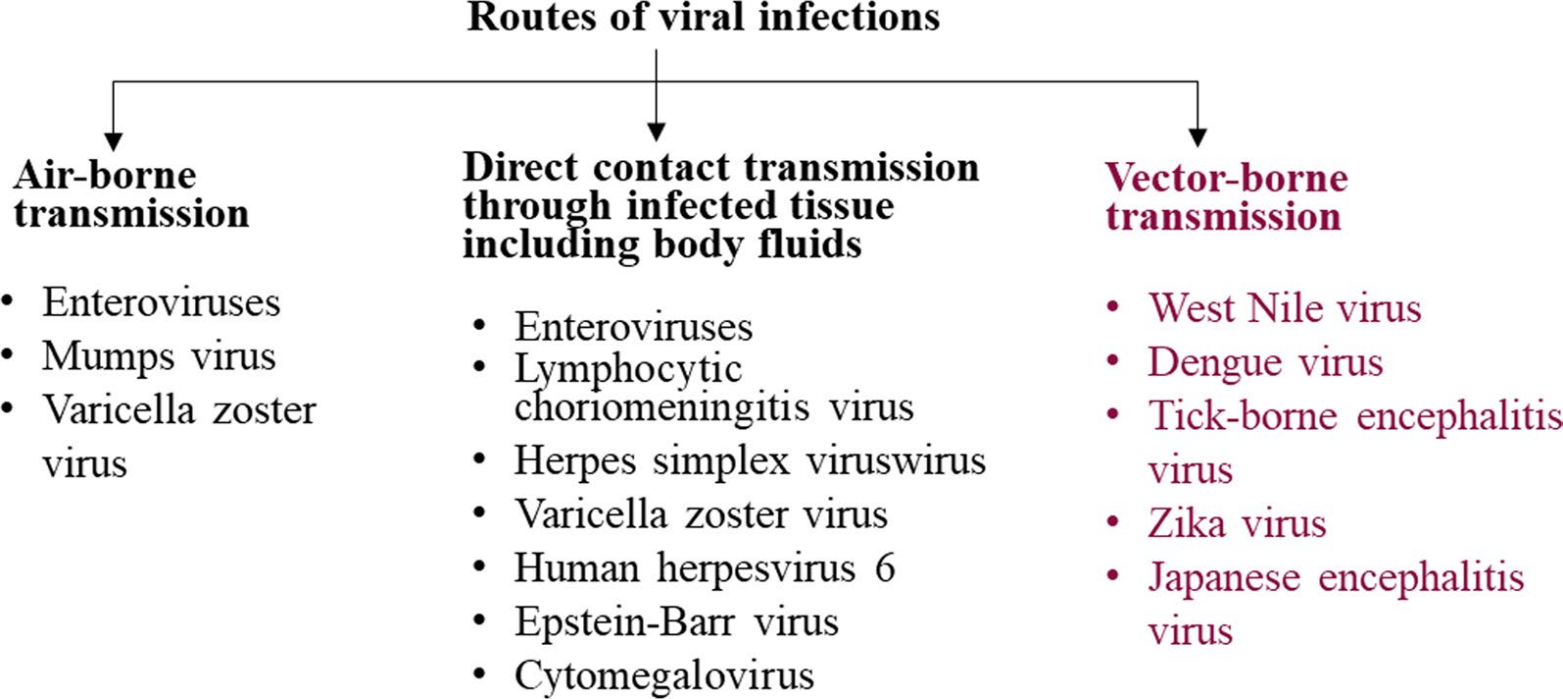
Different routes of transmitting viruses to the human body

Among diseases caused by external pathogens, viral infections pose the greatest number of diagnostic and therapeutic problems. Viruses that cause infections in humans enter the human body most often through by droplets or, direct contact, or can be transmitted by vectors (Fig. 1) [3].

### Viruses infecting the central nervous system

**Fig. 2:**
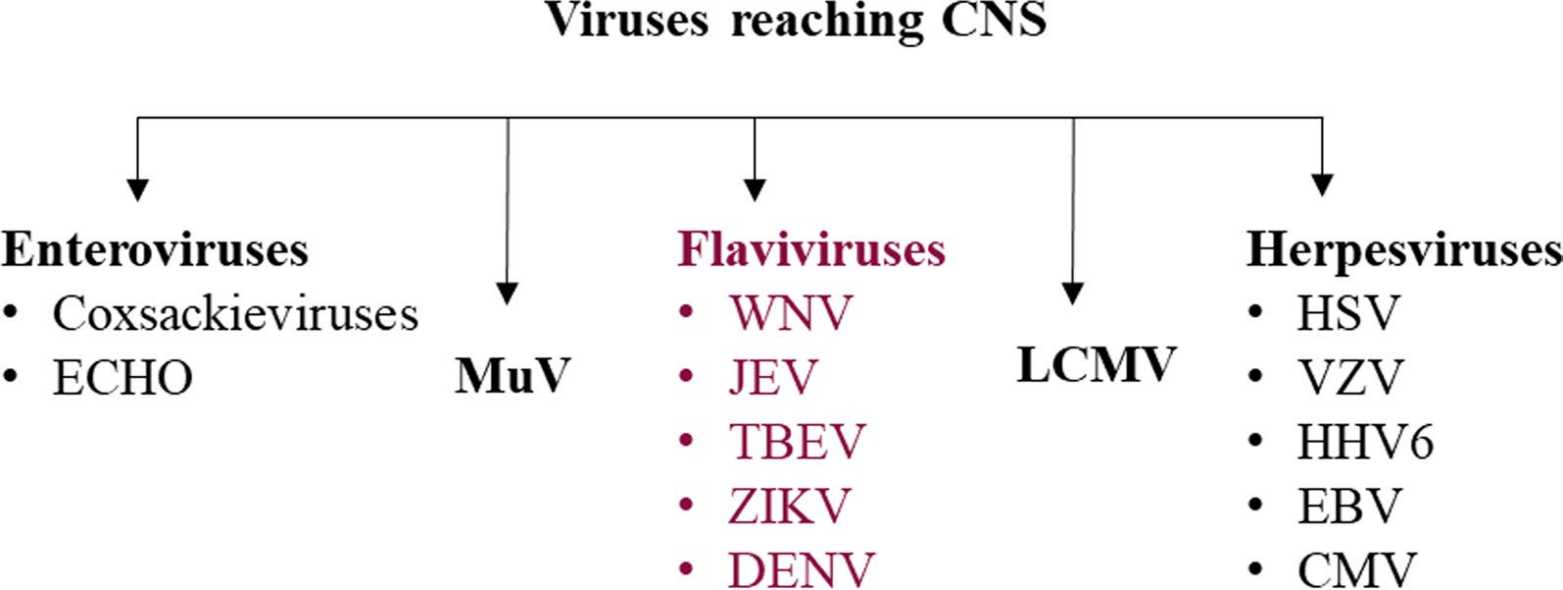
Viruses that reach the central nervous system (CNS)

Although most viruses replicate only in peripheral tissues, some have developed unique strategies to reach the central nervous system (CNS), where they cause infections [4]. As a consequence of a central nervous system infection, inflammation may occur in specific regions of the CNS, such as the meninges, brain, spinal cord, or simultaneously in many different regions of the CNS [4]. Viruses causing CNS infections mainly include enteroviruses [Coxsackie and ECHO], mumps virus (MuV), lymphocytic choriomeningitis virus (LCMV), flaviviruses [West Nile virus (WNV), Japanese encephalitis virus (JEV), tick-borne encephalitis virus (TBEV), Zika virus (ZIKV), and dengue virus (DENV)], and herpesviruses [herpes simplex virus (HSV), varicella-zoster virus (VZV), and human herpesvirus type 6 (HHV6)]; in people infected with human immunodeficiency virus (HIV), other members of the herpesvirus family [Epstein–Barr virus (EBV) and cytomegalovirus (CMV)] can become viral pathogens [5–17] (Fig. 2).

**Fig. 3:**
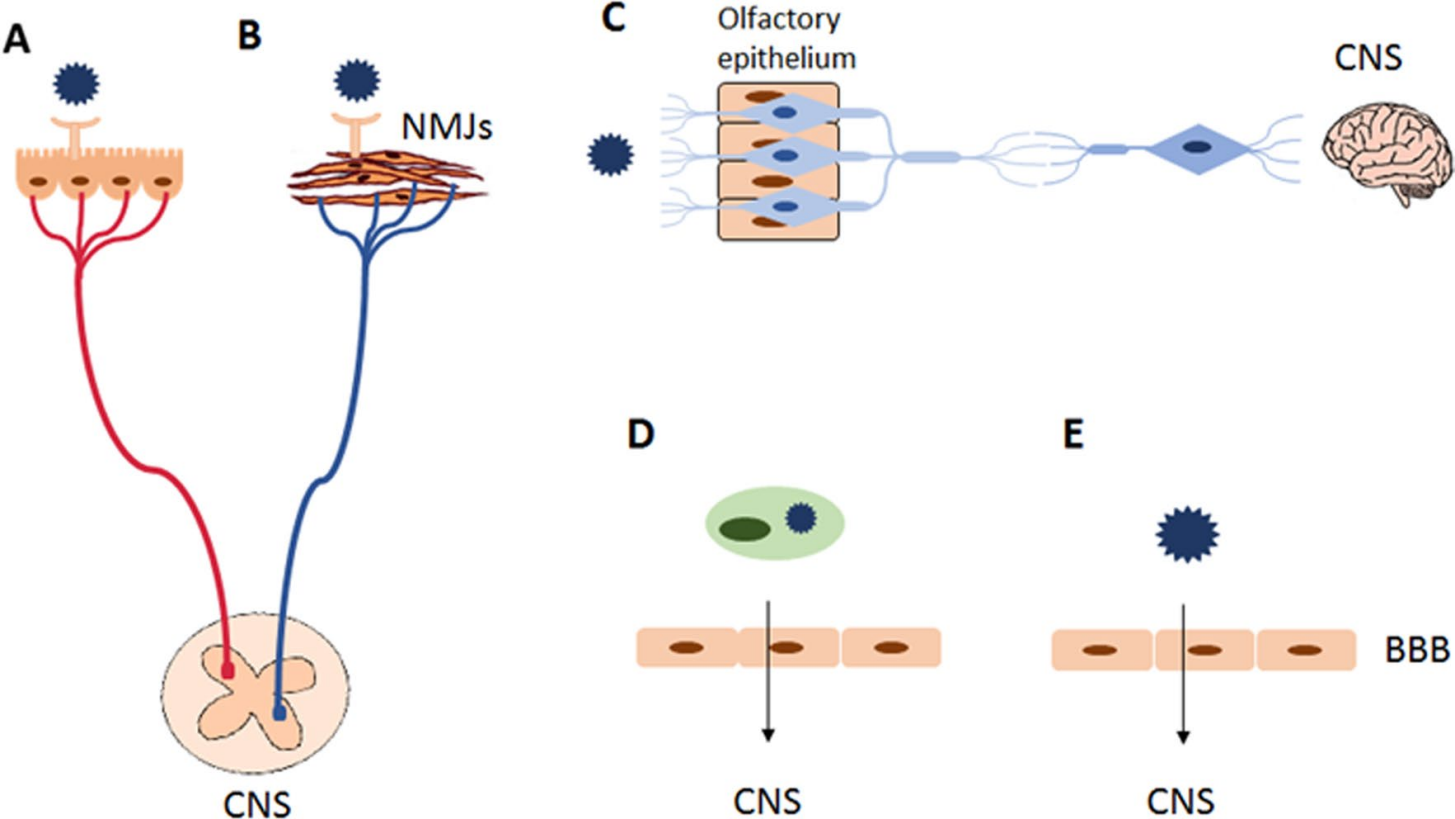
Virus Entry Routes into the CNS. **A** Spread via axons of sensory and autonomic neurons, **B** spread via neuromuscular junctions (NMJs), **C **infection of the olfactory epithelium, **D **trojan horse entry, **E **direct infection of endothelial cells (**EC**)

Viruses enter the peripheral nervous system (PNS) or the central nervous system (CNS) by infecting nerve endings in tissues directly or by infecting cells in the circulatory system, which carry them across the blood–brain barrier (BBB) to the CNS [18]. Herpesviruses can enter the PNS by binding to receptors on the axon terminals of sensory and autonomic neurons (Fig. 3A), which transmit sensory and visceral information. Most of these viruses use this route to enter the human body and establish a lifelong infection. Despite the direct synaptic connection between PNS neurons and the CNS, the spread of herpesvirus infection to the CNS is rare but has devastating effects [19]. Furthermore, the site of entry for viruses, such as rabies or poliovirus, into the CNS may be neuromuscular junctions (NMJs), since most motor neurons have their cell bodies in the spinal cord, which in turn are in synaptic contact with motor areas in the brain [20] (Fig. 3B). Rabies virus and poliovirus spread to the CNS via NMJs; the former enters the NMJ immediately after a bite from an infected animal, while the latter enters the NMJ by a more circuitous route. Viruses multiply in the mucosa of the gastrointestinal tract and then move to the lymph nodes and the blood; from there they can enter the CNS and replicate in motor neurons [20, 21]. It has been shown in animal models that viral invasion into the CNS can also occur via the olfactory epithelium and olfactory neurons, mainly in the case of HSV-1, vesicular stomatitis virus (VSV), Borna disease virus (BDV), RABV, influenza A virus, parainfluenza viruses, and prions (Fig. 3C) [22].

Moreover, some viruses gain access to the nervous system without infecting neurons, but rather by infecting leukocytes which, circulating in the blood, can penetrate the brain parenchyma. This mechanism is known as the ‘Trojan horse’ entry, as pathogens are hidden in those immune defense cells that are naturally able to cross the blood–brain barrier (BBB) (Fig. 3D) [18]. The BBB is the physiological barrier that separates the central nervous system (CNS) from the rest of the body and is crucial for the proper functioning of the brain and protecting the CNS from injury and disease. Its basic element are endothelial cells (ECs) of the capillaries, which are unique in the BBB—compared to ECs in other tissues—in that they are characterized by intercellular tight junctions (TJs) and do not contain fenestrae, i.e., small pores that allow intercellular transport by pinocytosis [23]. In addition, endothelial cells are characterized by low levels of transcytotic vesicles, which significantly limits both paracellular and transcellular movement of molecules through the EC layer. Furthermore, endothelial cells show low expression of leukocyte adhesion molecules (LAMs), which helps to limit the movement of immune cells from the blood to the brain. Due to intercellular tight junctions, CNS endothelial cells are characterized by unique properties specific only to the BBB, which ensure that the blood–brain barrier does not have the leakiness characteristic of peripheral endothelium [23]. Viral infections usually lead to a reduction in the expression and organization of tight junction proteins, directly affecting the integrity of the endothelial monolayer, which can result in cell death. However, viral replication in cerebral microvascular endothelial cells leads to increased production of leukocytes and cytokines, such as IL-6 and TNF-α, as well as of reactive oxygen and nitrogen species (ROS/RNS), which directly affects the structure of the BBB and may result in increased BBB permeability [24–26].

In some cases, viruses present in the circulatory system can also infect brain microvascular endothelial cells (BMVECs), a major component of the BBB (Fig. 3E). These viruses include West Nile virus (WNV), Epstein–Barr virus (EBV), human cytomegalovirus (HCMV), and mouse adenovirus 1 (MAV-1) [27–30]. Severe CNS infections can also be caused by paramyxoviruses, such as measles virus (MeV) and mumps virus (MuV) [6, 31]. Primary MeV and MuV infections start in the upper respiratory tract, with subsequent infection of lymphoid tissue resulting in viremia and spread of the virus to other tissues. MuV is highly neurotropic and can cause acute encephalopathy in children. Consequently, elevated levels of several cytokines, i.e., IFN-γ, IL-2, IL-6, and IL-10, are found in the cerebrospinal fluid of children diagnosed with MuV-related acute encephalopathy [32]. Unlike MuV, MeV infection spreads to the CNS in approx. 0.1 per cent of cases, causing several types of debilitating neurological diseases, including fatal subacute sclerosing panencephalitis (SSPE), which manifests weeks to years after infection [31, 33] (Table 1).

**Table 1:** Features of viruses infecting CNS and possibilities of preventing the disease

Features	Enterviruses	MuV	Flaviviruses	LCMV	Herpes-viruses
Genetic material of the virus	DNA	RNA	RNA	RNA	DNA
Presence of envelope	**−**	** + **	** + **	** + **	** + **
Route of infection	Food-borne, droplet direct contact	Droplet	Vector-borne	By rodents	Droplet direct contact
Symptoms	Most common from the skin and mucous membranes, less often from the muscles and nervous system	Typical: fever and swelling in the parotid gland area, rarely the nervous system	Nervous system infection	Nervous system infection	Can cause infection of the nervous system
Existing vaccines	Polio virus, HAV	**+ **	JEV, TBEV, DENV ZIKV—phase I clinical trials	−	VZV

The aforementioned selected examples of neuropathogenesis caused by different virus families show that although viral replication itself can be the cause, an activated immune system, in an attempt to eliminate the infection, can also contribute to neuronal damage. This review focuses on viruses of the *Flaviviridae* family.

Recruitment of peripheral immune cells into the CNS plays a fundamental role in the final outcome of neuroinfection caused by Flaviviruses, as T lymphocytes play the important role in the destruction of virus-infected cells, generation of cytokines, increasing the phagocytic activity of macrophages, and stimulation of local production of antibody by B lymphocytes [34]. De Vries et al. showed that during flavivirus infection, T cell subsets exhibit different migration patterns in the CNS. The majority of CD4 lymphocytes are retained in the perivascular spaces, while the majority of cytotoxic CD8 lymphocytes migrate into the parenchyma, where they perform their functions in the vicinity of infected neurons. In addition, cytokines and chemokines recruit leukocytes that can induce BBB breakdown. In addition, flaviviruses stimulate leukocytes to secrete proteases affecting the integrity of the BBB. Moreover, it was found that JEV and DENV induce the secretion of serine protease (chimases) by mast cells, which degrades BBB proteins and simultaneously destroys its structure [35, 36].

### Metabolic changes as a response to viral infection

Under physiological conditions, the brain, in a manner similar to other organs and tissues of the body, is characterized by redox homeostasis resulting from the balance between the production of reactive oxygen species (ROS) and the action of antioxidant mechanisms. On the other hand, brain cells are thought to be particularly susceptible to a shift in this balance towards pro-oxidant conditions due to their consumption of relatively large amounts of oxygen for energy production and weaker antioxidant defense mechanisms, compared to cells in other organs, which favors the development of oxidative stress. Thus, even under physiological conditions, the brain has a high pro-oxidant potential and, at the same time, high metabolic requirements, which makes it particularly susceptible to oxidative stress [37]. In addition, the cell membrane of neurons contains high concentrations of polyunsaturated fatty acids (PUFAs), which are particularly susceptible to oxidative modification by ROS [38], which, as a consequence, may interfere with the proper functioning of these cells under metabolically altered conditions, including those resulting from infection [19].

Viral infections are usually initiated in the periphery, mainly in the epithelium or endothelium of cells. As a consequence, an immune response and paracrine signaling occur, which are initiated in infected cells and transferred to uninfected cells by secreted cytokines [39]. In contrast, the virus can be cleared by the action of specific antibodies to the virus and T lymphocytes as part of the adaptive immune response [34]. In a different situation, however, viral infection can spread to other tissues, leading to a strong systemic immune response, including after getting through the blood–brain barrier, it can cause devastating effects in the central nervous system (CNS) [19]. Therefore, viral infections of the nervous system induce a complex multicellular response involving communication between multiple cell types that serves to minimize virus spread, clear virus and cell debris, protect host cells, and preserve neuronal function. Viral replication in neurons and the subsequent local inflammatory response are responsible for the neuropathogenesis of infection with viruses reaching the brain, including flaviviruses [39].

The consequence of CNS infection is, therefore, inflammation and accompanying oxidative stress, caused mainly by mitochondrial dysfunction associated with increased mitochondrial complex I activity and increased production of superoxide anion radicals as a result of disruption of the electron transport chain and their leakage from the mitochondrial matrix, which can catalyze the autoxidation of hemoglobin and free-radical enzymatic reactions, which in the context of high concentrations of iron ions, as a component of heme, leads to the formation of hydrogen peroxide and highly reactive hydroxyl radicals, which in turn can further increase the level of neural tissue damage by inducing oxidative damage to neurons [40]. Furthermore In astrocytes and microglia, this is accompanied by an increase in the secretion of nitric oxide and pro-inflammatory cytokines, such as IL-6 and TNF-α [41]. Moreover, infection (under conditions of oxidative stress) additionally results in the activation of pro-oxidant enzymes, including NADPH oxidase and xanthine oxidase, which produce large amounts of superoxide anion radicals and nitric oxide synthase responsible for NO generation [42]. Under physiological conditions, antioxidant enzymes metabolize ROS; however, excessive production of ROS resulting from infection may prevent their effective elimination [43]. In addition, viral infections usually contribute to a decrease in the efficiency of antioxidant enzymes by reducing the availability of copper and zinc ions, among others, consequently reducing superoxide dismutase (Cu, Zn-SOD) activity [44]. Under physiological conditions, superoxide dismutase is responsible for the dismutation of superoxide anion radical, resulting in the formation of hydrogen peroxide, which is removed by glutathione peroxidase, thus preventing the formation of hydroxyl radicals. However, under pro-inflammatory conditions resulting from infection, higher superoxide dismutase activity relative to glutathione peroxidase is observed in patients’ plasma, leading to increased levels of hydroxyl radicals and enhanced oxidative processes with consequent modification of cellular components, resulting in disruption of metabolic pathways and signaling in brain cells [45].

Viral infections of the CNS can cause a severe course of the disease with accompanying short- and long-term sequelae and mortality. Viral infection of the CNS can lead to inflammation of the meninges lining the brain (meningitis), the brain itself (encephalitis) and the spinal cord (myelitis) [4]. Neurotropic viruses are able to cross the blood–brain barrier to attack the central nervous system and cause disease through virus-induced cytopathology or neurotoxic antiviral immune response of the patient's body [18].

During viral infections, metabolic changes are observed in the host organism, which both enable the replication of the virus and those whose task is to protect the organism against the consequences of infection [46]. The vast majority of these activities take place at the mitochondrial level, because energy in the form of ATP is required to introduce the virus into the cytoplasm of the host cells via the receptor. In contrast, viruses, as absolute intracellular parasites, are completely dependent on the metabolic mechanism of the host cell to provide the energy and compounds necessary for their replication. It is believed that up-regulation of various elements of glycolysis, including the rate of glycolysis by facilitating nucleotide synthesis, can promote viral replication [47], making the TCA cycle a key element in the biosynthesis of compounds needed for viral replication to occur in host cells at the level of the mitochondrial matrix, which in turn provides a precursor, such as citrate for fatty acid synthesis. In addition, viruses can reprogram the TCA cycle by enhancing the biosynthesis of fatty acids, including long-chain fatty acids required for the formation of viral membranes [48]. In addition, it is known that viruses can co-opt TCA cycle metabolites for post-translational modifications of viral proteins, including acetyl-CoA can serve as a substrate for lysine acetylation of the nuclear antigen (LANA) encoded by Herpes virus [49]. It has also been shown that a glycolytic pathway is induced in DENV infection to promote efficient viral replication [50], resulting in an increase in glucose consumption and expression of glucose transporter 1 (GLUT1) and hexokinase 2 (HK2), and inhibition of this metabolic pathway reduces DENV replication [50]. The increase in glucose consumption may also be due to its use by DENV-infected cells to increase their ability to oxidize endogenous or exogenous fatty acids [51]. Virus replication requires adequate levels of ATP provided by B-oxidation [52]. Another major carbon source used to meet the energy needs of mammalian cells is glutamine, which supports the TCA cycle. Extensive reprogramming of carbon metabolism has also been observed during viral infection. HCMV-infected cells have been found to use glutamine to fuel the TCA cycle, allowing carbon from glucose metabolism to be used for fatty acid synthesis [53]. In addition, glutamine is consumed in a number of metabolic pathways that supply nitrogen for nucleotide biosynthesis. It has been found that the inhibition of DENV replication under glutamine-deficient conditions may be related to the need to utilize an increased intracellular pool of nucleotides during viral replication, and thus the levels of compounds involved in purine and pyrimidine metabolism are significantly elevated in DENV-infected cells. Therefore, it has been suggested that DENV, to meet its replication needs, may require glutamine as both a carbon and nitrogen source [50]. Consequently, flaviviruses modify host cellular metabolism by increasing the pool of nucleotides and enzyme cofactors, such as ATP for RNA helicase activity [54]. This promotes a change in the structure of the host's endoplasmic reticulum membrane to establish protected replication sites. These replication compartments are thought to promote appropriate replicase scaffolding and concentration of replication substrates, and play a protective role for viral RNA against cytosolic sensors of innate immunity and its degradation mechanisms [54].

Viruses can also interfere with fatty acid oxidation, which is a catabolic process in which phospholipid fatty acids are metabolized to produce energy. Fatty acid oxidation has been found to be crucial for measles virus proliferation [55], while DENV infection also induces lipid degradation and enhances β-oxidation, and etomoxir (β-oxidation inhibitor) therapy reduces DENV replication [52]. In addition, dengue virus studies have shown that DENV NS4B induces mitochondrial elongation by inactivating dynamin-related protein 1, which ultimately weakens the host's antiviral immune response [41]. Given that Zika virus exhibits similar effects, it has been suggested that promoting mitochondrial elongation may be a common strategy specific to flaviviruses [56].

However, in recent years, it has been increasingly emphasized that mitochondrial metabolism is also a key element in the prevention of viral infection, indicating that mitochondrial antiviral signaling, which through the tricarboxylic acid (TCA) cycle, electron transport of the respiratory chain complex and fatty acid oxidation, is an essential component of the host immune response [56]. Metabolites of the TCA cycle can serve as substrates to enhance human defense mechanisms, including acetyl-CoA is a cofactor required for the expression of IFN-γ in effector T cells [57], fumarate inhibits pyroptosis in macrophages by reacting with GSDMD via Michael addition reaction [58], while conversion of the immune response gene cis-aconitrate 1 (IRG1) to itaconate induces expression of anti-inflammatory genes to counteract pro-inflammatory responses [59]. At the same time, both itaconate and fumarate can strongly inhibit viral replication through mechanisms that have not been clearly identified to date.

Itaconate is known to inhibit succinate dehydrogenase, thereby regulating succinate levels, mitochondrial respiration and the production of pro-inflammatory cytokines (IL-1β, IL-6, IL-12) [60, 62], and induces electrophilic stress and inhibits inflammation mediated by IκBζ [63], up-regulating the expression of secondary response genes that enhance TLR/IL-1R signaling pathways leading to cytokine production [64, 65]. Itaconate, by modifying the structure and function of proteins on cysteine residues, acts as an immunomodulator [66]. In addition, the itaconate derivative 4-octyl-itaconate (4-OI) has been shown to reduce the host inflammatory response associated with infection, while inhibiting replication of Zika virus, among others [67]. Thus, itaconate and its derivatives reduce inflammation and associated pathologies and inhibit viral replication. Using a mouse model of ZIKV infection, ZIKV was found to activate a signaling pathway involving receptor-interacting protein kinase-3 (RIPK3), leading to the up-regulation of Acod-1, which inhibits ZIKV replication in neurons [68]. In addition, by alkylating the cysteine residues of the Keap1 protein, an inhibitor of the cytosolic transcription factor Nrf2, itaconate prevents its degradation and enables its nuclear translocation and transcriptional efficiency against antioxidant and anti-inflammatory proteins [69, 70]. Nrf2 is also activated by the succinate oxidation product fumarate and its derivatives, such as monomethyl fumarate (MMF) and dimethyl fumarate (DMF), which are both potent immunomodulators and antioxidants [71]. However, it has recently been shown that expression of the Nrf2 gene, which regulates transcription of the glutathione and thioredoxin antioxidant systems, detoxification, NADPH regeneration and heme metabolism [72], is reduced in biopsies obtained from COVID-19 patients [67], and it is known that in addition to its antioxidant and anti-inflammatory effects, Nrf2 can also regulate the detection of viral DNA in the cytoplasm and thus release the antiviral type I IFN [73]. Fumarate, a product of succinate oxidation by succinate dehydrogenase, and its derivatives monomethyl fumarate (MMF) and dimethyl fumarate (DMF) are potent immunomodulators and antioxidants that activate Nrf2 [74, 75]. DMF inhibits the maturation of dendritic cells (DCs) [76] and drives the production of IL-10, IL-12 and IL-23 by DCs, thereby reducing pathogenic T cells [77], DMF also inhibits Th1 to Th2 cell transitions, pro-inflammatory cytokine signaling and nuclear translocation of the pro-inflammatory transcription factor NF-κB and expression of adhesion molecules in lymphocytes and endothelial cells [78, 81].

Byproducts of the mitochondrial electron transport chain are ROS [82], which can in excess generate deleterious effects, such as oxidative modifications of lipids, proteins and nucleic acids. Increasingly, ROS have been identified as active elements in intercellular signaling [83], including those involved in immune defense against viruses and other pathogens [84]. Cellular sources of ROS include NADPH oxidase and the mitochondrial respiratory chain [85]. In addition, nascent ROS through activation of inflammasomes, including NLRP3, are involved in innate immunity, among other things [86]. It has even been suggested that reducing the level of ROS in mitochondria can increase viral replication [87]. An important component of mitochondrial metabolic activity is that these cellular organelles do not function in isolation, but in interaction with other organelles, such as the endoplasmic reticulum (ER), which is particularly important for lipid biosynthesis. In addition, subdomains of mitochondrial–ER connections have been found to be important in the induction of antiviral signaling involving the mitochondrial MAVS protein, allowing, among other things, the detection of viral RNA [88].

It is known, that the increased energy and lipid metabolism essential for the replication of many viruses is reversed by IFN, acting to control viral infections. One of the interferon stimulated genes induced by type I IFN, affects cellular metabolism and encodes the enzyme cholesterol-25-hydroxylase (CH25H), which converts cholesterol to soluble oxysterol 25-hydroxycholesterol (25HC) that in turn serves to decrease cholesterol accumulation within cells. The overall effect is increased resistance to several viruses, such as ZIKV and other flaviviruses [89].

In the course of flavivirus neuroinfections, microglia can physically surround and phagocytose dying neurons. The reactive microglia can eliminate the dysfunctional synapses, what most likely serves a neuroprotective function. The knowledge about the mechanisms of microglial neuroprotection in viral meningitis and encephalitis may help to design rational targeted therapeutic possibilities. Another mechanism involved in neuroinfections include changes of astrocytes in morphology, gene expression, proliferative capacity, and function (reactive astrogliosis) [90].

All the above facts indicate that virus–host interactions at the cellular level are not simple and unambiguous. The consequence is a constant search for answers as to whether there is an unambiguous way to prevent the effects of virus–host interactions.

### Flaviviruses

Flaviviruses, which are RNA viruses, are the one group from the *Flaviviridae* family that is widespread globally. The most common arthropod-borne flaviviruses include WNV, DENV, TBEV, ZIKV, and JEV (Fig. 2). They have a high affinity for CNSs by which they can cause a number of potentially fatal, serious diseases, including encephalitis, acute flaccid paralysis or fetal birth defects. In recent years, a surge in the number of infections caused by flaviviruses, such as dengue virus, West Nile virus, and Zika virus, in particular, has been observed, with epidemics occurring in the Americas, among other places (WHO: https://www.who.int/news-room/fact-sheets/detail/west-nile-virus, CDC: https://www.cdc.gov/vhf/virus-families/flaviviridae.html).

Flaviviruses are enveloped viruses that have a single-, positive-stranded RNA genome containing a 5′ cap and 5′ and 3′ untranslated regions (UTRs), as well as a single open reading frame (ORF). The ORF encodes a large polyprotein that is degraded co-translationally and post-translationally into three structural proteins, i.e., C (capsid protein), prM (pre-membrane protein), and E (envelope protein), as well as seven non-structural proteins (NS1, NS2A, NS2B, NS3, NS4A, NS4B, and NS5). The virus particle consists of structural proteins that play an important role in its entry into the host cell, as well as in the assembly and release of new virions. In addition, the capsid protein binds genomic RNA to form the core of the nucleocapsid, while glycoproteins E and prM are viral surface proteins attached to the host-derived lipid envelope. Non-structural proteins, on the other hand, form a viral replication complex inside the host cell [91, 92].

Flavivirus infection occurs mainly through a host being bitten by a mosquito or a tick, leading to an infection of macrophages and dendritic cells (DCs) in the dermis [93]. After local proliferation of the virus, infected dendritic cells can transport the virus to the lymph nodes, allowing it to spread throughout the body, reaching various organs, including the brain [93]. When the virus gains entry to the CNS, the innate immune response is the first line of host defense. It is initiated by pathogen-associated molecular patterns (PAMPs) with the involvement of pattern recognition receptors (PRRs). PRRs, including RIG-I-like receptors (RLRs), e.g., RIG-I and MDA5 and Toll-like receptors (TLRs), recognize RNA and viral proteins and activate transcription factors, i.e., interferon regulatory factors 3 and 7 (IRF3, IRF7) and NF-κB, leading to the production of type I interferons (IFN-α/β) and pro-inflammatory cytokines (IL-6 and IL-8), causing inflammatory and stress responses [26, 94]. The released IFN-I molecules bind to IFN-I receptors and activate the JAK/STAT signaling cascade, which drives the expression of a wide range of interferon-stimulated genes responsible for the induction of antiviral gene expression and increased production of inflammatory (IL-6) and immunoregulatory (IL-4, IL-10) cytokines [95]. This causes flaviviruses to attempt to inhibit JAK/STAT signaling to disturb the cellular response to interferon and cytokines [96]. Viral components and cellular metabolites produced by viral replication can also stimulate elements of the inflammasome complex, leading to secretion of pro-inflammatory interleukin IL-1β and, ultimately, to cell death [97]. The arising pro-inflammatory conditions are also favored by the activation of the NF-κB signaling pathway, which induces pro-inflammatory genes, including those encoding tumor necrosis factor (TNF-α), i.e., IL-1 and IL-6 [98]. Signaling by NF-κB depends on interaction with the non-enzymatic cellular antioxidant, GSH, which, by modifying the structure of NF-κB through glutathionylation, inhibits its pro-inflammatory effects [99]. However, infections with certain flaviviruses have been found to reduce GSH levels [45, 100], which may enhance the development of inflammation and viral replication.

Due to the fact that inflammatory conditions are usually accompanied by oxidative stress, increased generation of reactive oxygen species (ROS), including superoxide anion radical, hydroxyl radical, and hydrogen peroxide, is observed in flavivirus-infected cells [101]. At the same time, a reduction in antioxidant capacity at the level of enzymatic antioxidants is observed, i.e., superoxide dismutase (SOD), catalase, and glutathione peroxidase (GPX) activity, whose biosynthesis is dependent on the transcriptional activity of the Nrf2–Keap1 pathway [98]. Nrf2 is sequestered in the cytosol by interacting with Keap1, facilitating Nrf2 ubiquitination and its proteasomal degradation, thereby limiting the expression of Nrf2-regulated genes. However, under oxidative stress conditions, the Keap1 conformation is modified, mainly through interaction with 4-hydroxynonenal (4-HNE), a product of arachidonic acid peroxidation, resulting in the release of Nrf2 from the complex with Keap1 into the cytosol and its transfer to the cell nucleus, where Nrf2 forms a heterodimer with small Maf proteins (sMaf) and binds to the antioxidant response element (ARE). This initiates ARE-dependent transcription of cytoprotective proteins [102], including antioxidant enzymes, to attenuate cellular oxidative stress and combat viral infection. However, some flaviviruses (DENV, ZIKV) inhibit the transcriptional activity of Nrf2, leading to inhibition of transcription of antioxidant genes, which consequently contributes to a shift of the redox balance towards oxidative conditions and the development of infection [44, 103]. Regardless of the effectiveness of antioxidant proteins, host cells are equipped with small-molecule antioxidants, such as vitamin C, vitamin E, and glutathione (GSH), whose levels are also significantly reduced by flavivirus infection [45]. Consequently, increased ROS production and reduced antioxidant defences lead to increased viral infection.

### West Nile Virus

West Nile Virus (WNV) is a mosquito-borne flavivirus whose natural hosts are birds, while humans and horses are its accidental hosts (WHO: https://www.who.int/news-room/fact-sheets/detail/west-nile-virus). WNV is endemic in various areas of Africa, Asia, and the Middle East. Moreover, it has recently been identified by the Centers for Disease Control and Prevention (CDC) as the leading cause of mosquito-borne disease in the continental states of the USA (CDC-https://www.cdc.gov/westnile/statsmaps/index. html). By January 10, 2023. a total of 1,035 cases of illness due to West Nile virus infection in humans had been reported to the CDC, of which 737 cases (71%) were classified as neuroinvasive disease (including meningitis or encephalitis) and 298 cases (29%) indicated infection of the non-invasive type (https://www.cdc.gov/westnile/ statsmaps/preliminary mapsdata2022/index.html). Those individuals who are extremely susceptible to WNV infection, including the elderly, chronically ill, and/or immunocompromised patients, can develop severe encephalitis and are more prone to death as a result of the disease [104].

**Fig. 4:**
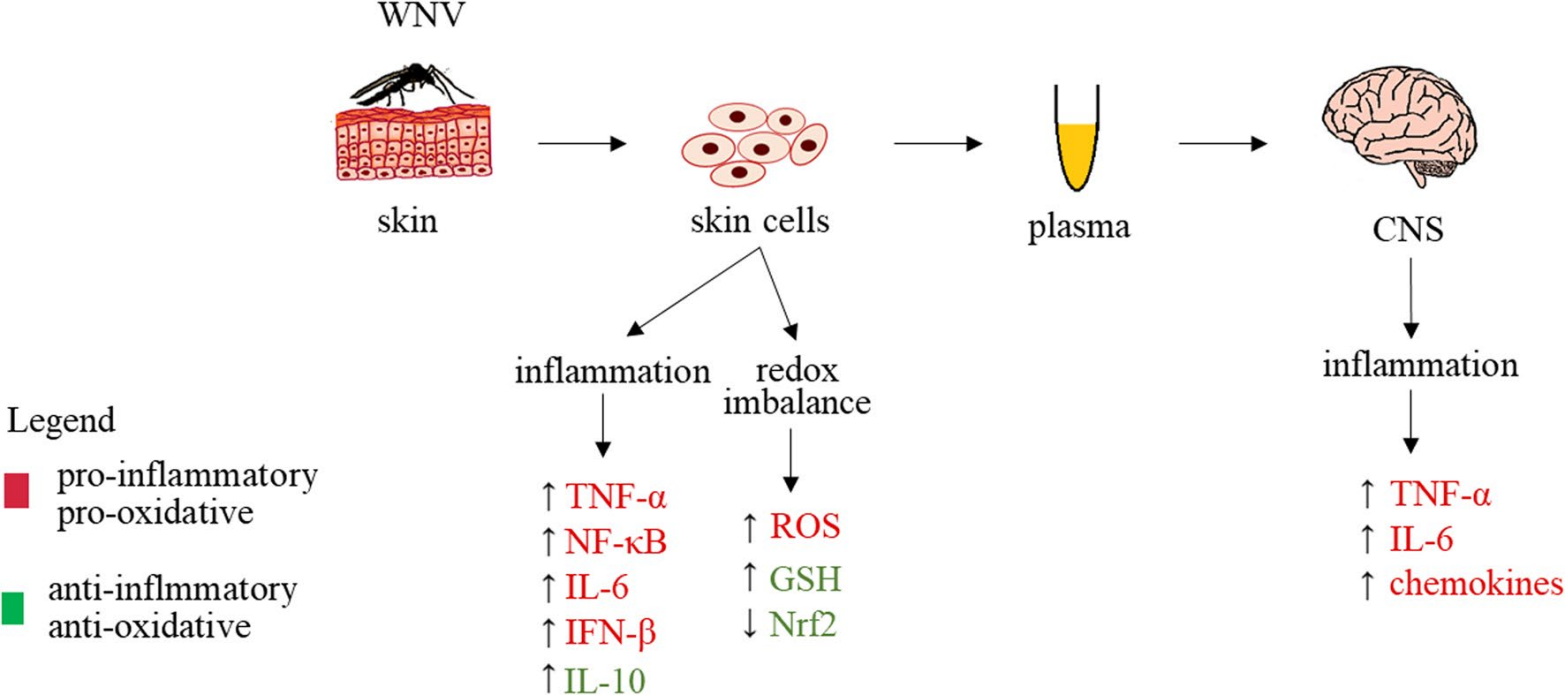
Metabolic changes in cells and the CNS during West Nile Virus (WNV) infection

Introduced into the human body by a mosquito bite, WNV enters skin cells and then the blood. It has been suggested that the virus infects resident dendritic cells in the skin, such as Langerhans cells, which then travel to the draining lymph node [105]. In this case, infection and the risk of viral spread are counteracted by the rapid development of the early immune response, including the production of IFN-β and IFN-γ and the effector functions of innate immune cells (γδ T cells, NK cells, neutrophils, and macrophages) [106, 109]. Infected human cells detect the virus and induce IFN-β production through the recognition of viral RNA by retinoic acid-inducible gene I (RIG-I) and melanoma differentiation-associated gene 5 (MDA5) [110]. The binding of viral RNA promotes interaction with interferon-beta promoter stimulator 1 (IPS-1), which results in the recruitment of signaling proteins (e.g., NEMO and TRAF3), leading to the activation of interferon regulatory factor 3 (IRF-3) and NF-κB. These factors travel to the nucleus and bind to the promoter region of the IFN-β gene, with IFN-β production leading to a reduction in viral infection [111]. IFN-β is the first line of host defense against viral infection, so West Nile virus, mediated by nonstructural protein NS1, inhibits the expression of IFN-β. NS1 through direct interaction with RIG-I and MDA5 causes their degradation. This in turn leads to the inhibition of IFN-β expression, preventing further activation of the RLR signaling pathway [112]. WNV, via the Toll-like receptor, induces secretion of pro-inflammatory cytokines, including IL-6 and TNF-α, in peripheral tissues [113]. This suggests that secreted TNF-α may also modulate BBB permeability by altering the tight junctions of endothelial cells, allowing WNV to penetrate the BBB and infect neurons [113]. The mechanism by which WNV penetrates the BBB and causes brain inflammation remains unexplained. The activation of pattern recognition receptors (PRR) in the BBB endothelium by WNV results in cytokine-dependent increase in BBB permeability, which results in the virus entering the CNS [114]. WNV can also penetrate the CNS by other mechanisms, e.g., retrograde axonal transport, and then spread to the neuronal level [115]. Another possible mechanism of WNV entry into the CNS is the ‘Trojan horse’ mechanism, whereby the virus is transported by infected immune cells, such as lymphocytes or neutrophils [106, 116]. The neuroinflammatory response to WNV infection includes strong activation of microglia and astrocytes, which also leads to the release of pro-inflammatory cytokines, such as TNF-α and IL-6, and chemokines, such as chemokine (C–C motif) ligand 2 and 5/CC-chemokine ligands 2 and 5 (CCL2, CCL5) and C–X–C motif chemokine 10 (CXCL10) [117]. These pro-inflammatory mediators also promote the recruitment of peripheral immune cells to the CNS following viral infection and regulate their function, controlling the proliferation and removal of the virus [118–120]. An increase in the pro-inflammatory response during WNV infection may also enhance the secretion of low levels of IL-10, an anti-inflammatory cytokine, by dendritic cells [121]. WNV infection has also been shown to induce an increase in reactive oxygen species (ROS) generation in infected hamster kidney cells. This increase in ROS generation results in an increase in GSH levels, leading to a new level of cellular homeostasis (Fig. 4) [122].

### Dengue virus

Dengue virus (DENV) is a flavivirus transmitted by mosquitoes of the genus Aedes which causes one of the most problematic arthropod-borne viral infections in the world. It reveals itself as a self-limiting febrile illness that may lead to death. The incidence of dengue is growing faster than that of any other infectious disease, with a dramatic increase seen in recent decades. According to the WHO, over the past two decades, the number of reported cases of dengue has increased more than 8 times, i.e., from 505,430 to 5.2 million cases. The highest incidence rates are seen in Asia (75%), Latin America, and Africa (WHO: https://www.who.int/health-topics/dengue-and-severe-dengue/dengue---timor-leste#tab=tab_2).

Most cases of dengue are asymptomatic or characterized by mild symptoms. However, it can also manifest as a severe flu-like illness characterized by high fever, severe headache, muscle and joint pain, nausea, vomiting, enlarged lymph nodes, and a rash. Moreover, some people may develop a severe form of dengue fever, which can cause bleeding, organ damage, and even lead to death (WHO: https://www.who.int/news-room/fact-sheets/detail/dengue-and-severe-dengue, [123]).

After a person is bitten by a DENV-carrying mosquito, dendritic cells (DCs) and skin Langerhans cells are the first to become infected; through them, the virus then reaches the lymph nodes [93] containing target cells, such as monocytes, macrophages, and lymphocytes [124]. Dendritic cell genes, such as RIG-I and MDA5, have been found to induce the secretion of IL-1β, IL-6, and TNF, as well as CCL2, CCL3, and CCL4, in response to products of DENV RNA replication [125]. Increased IL-6 production has also been found in children hospitalized for dengue virus encephalitis [126]. Five serotypes of dengue virus are currently known, i.e., DENV 1, DENV2, DENV3, DENV4, and DENV5 [127, 128]. Infection with any one of them provides permanent protection against the same virus strain, while secondary infection with another strain can cause the pathological phenomenon known as antibody-dependent enhancement (ADE), which leads to severe symptoms of the disease. The reason for this is that antibodies produced during the first infection bind to the virus of another strain, but do not neutralize it, while antibody-coated non-neutralizing DENV facilitate entry into phagocytic cells promoted by overexpression of Fcγ receptors [129]. Moreover, T cells from previous DENV infections may contribute to the ineffectiveness in eliminating virus-infected cells, as may the secretion of cytokines, such as IL-6 and IL-10, while IL-12 and IFN-γ levels are down-regulated and signals triggered by cytokine–receptor interactions activate STAT-1 and IRF-1. This results in the activation of iNOS gene transcription and enhanced nitric oxide (NO) generation, leading to strong anti-DENV suppression by ROS. This situation is observed especially in severe secondary infection [130]. It has been shown in in vitro studies that inhibition of the activity of dengue virus non-structural protein NS5 by nitric oxide leads to a decrease in DENV replication [131]. Thus, it has been suggested that NO acts as an important immune mediator against dengue virus infection and its levels may be regulated by it. However, in ADE infection, the levels of IL-12 and IFN-γ are reduced, leading to increased expression of IL-10, which acts as an autocrine factor and binds to a specific receptor causing the inhibition of STAT-1 and IRF-1 activation, thus reducing NO generation [130]. However, another non-structural dengue virus protein, NS1, modulates dengue pathogenesis by directly activating monocytes/macrophages to secrete cytokines that, by disrupting endothelial integrity, increase endothelial permeability, leading to hemorrhage [132].

The immune response to DENV infection begins with the production of antibodies, followed by secretion of type I IFNs, cytokines, and chemokines (CXCL10, CXCL11, IL-6, CCL3, CCL5) [133, 134] to present the antigen to T cells. On the other hand, NOX-dependent oxidative stress has been found to activate, as part of its antiviral effect, regulatory factors IRF-3 and IRF-7, signal transducer and activator of transcription 1 (STAT-1), and transcription factor NF-κB in B cells, which is associated with severe damage to virus-infected cells [135]. Furthermore, high levels of circulating pro-inflammatory cytokines such as IL-1 β or TNF-α found in the bodies of patients also correlate with a more severe disease course in DENV-infected patients [136]. Such a response is favored by nonstructural protein NS2B, which increases viral replication and the expression of inflammatory or apoptotic genes, leading to a progressive increase in inflammation and death of infected cells [103].

**Fig. 5:**
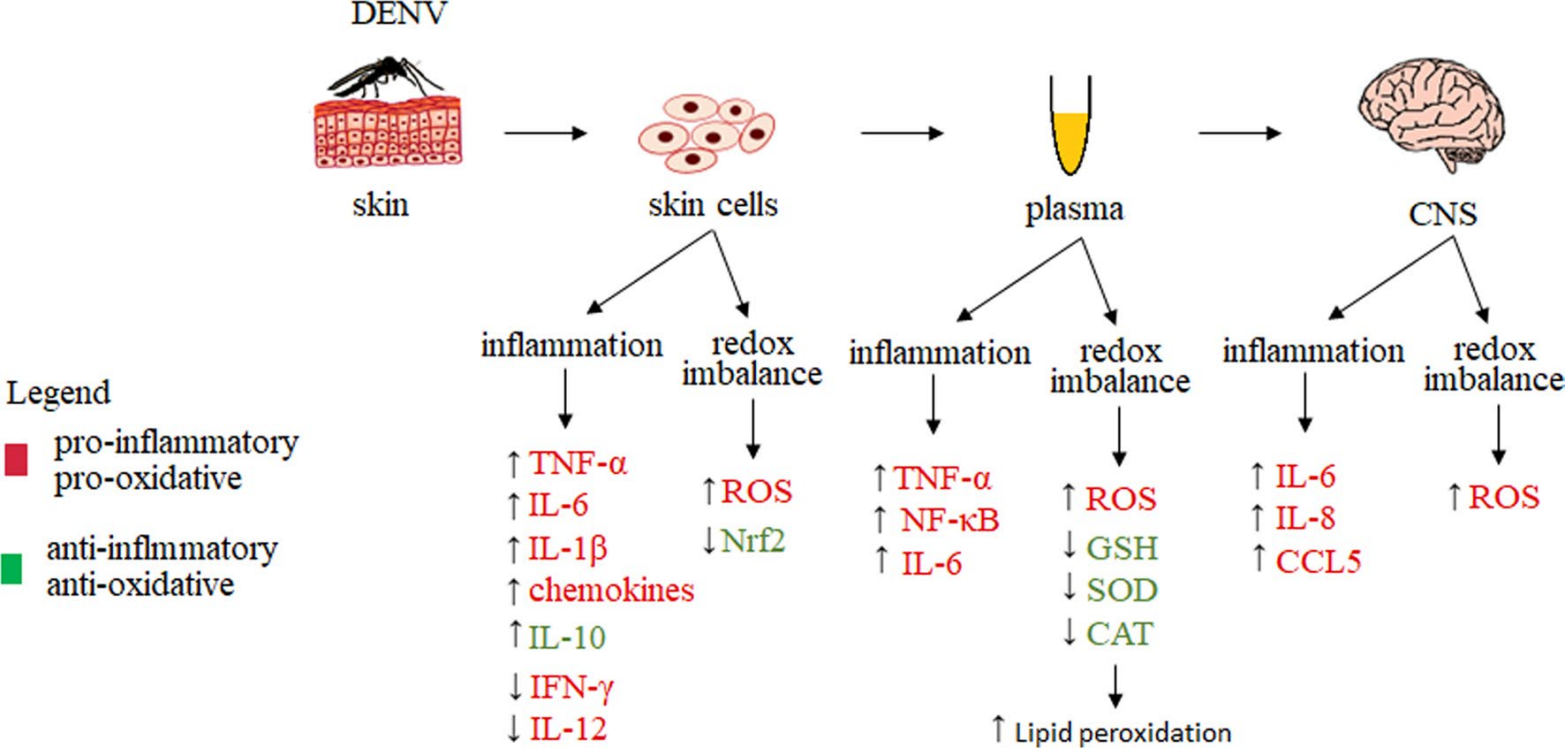
Metabolic changes in skin cells (in vivo and in vitro), blood and the CNS during Dengue Virus (DENV) infection

Consequently, both oxidative stress and innate immune response determine the severity of dengue disease. Infection of human brain microvascular endothelial cells (HBMEC) with dengue virus has been shown to activate NADPH oxidase, which enhances the generation of ROS. By increasing the secretion of chemokines and inflammatory cytokines (CCL5, IL-6 and IL-8), this increases viral replication and induces cell death, thus contributing to increased endothelial permeability [137]. In addition, by reacting with host cell components, ROS cause, e.g., an increase in lipid peroxidation with an increase in malondialdehyde (MDA) levels observed in patients’ plasma [138]. These findings indicate an unequivocal relationship between inflammation and oxidative stress and the development of a severe form of dengue [138]. Moreover, parallel activation in dendritic cells of antioxidant pathways regulated by the transcription factor Nrf2 could, by attempting to maintain redox homeostasis, contribute to the control of antiviral and apoptotic responses [135]. However, in vitro studies on human monocyte-derived dendritic cells have shown that dengue virus, similar to other members of the *Flaviviridae* family, can use nonstructural viral proteins to disturb or degrade critical signaling components to circumvent the antiviral response. Nonstructural protein NS2B has been found to reduce Nrf2 activity, leading to the inhibition of antioxidant protein genes and a progressive increase in ROS levels [103]. This is supported by studies in DENV-infected mice showing that, during infection, an increase in ROS levels with a concomitant decrease in intracellular GSH, SOD, and CAT activity can be observed, resulting in oxidative stress with increased lipid peroxidation, assessed via MDA levels and NF-κB activation. This resulted in increased TNF-α and IL-6 levels in the serum of DENV-infected mice, which enhances dengue virus replication [139]. On the other hand, supplementation of mice with glutathione was found to lead to enhanced antioxidant capacity by increasing SOD and CAT activity and inhibiting lipid peroxidation, as well as decreasing NF-κB activation with reduced secretion of pro-inflammatory factors, including TNF-α and IL-6 (Fig. 5) [139, 140].

The effect of dengue virus on metabolic processes in target cells is of interest. This virus uses the lipid reserves of the host cell, which are stored in lipid droplets, which are degraded to release fatty acids using an autophagy-type mechanism. These acids undergo oxidation, which drives the tricarboxylic acid cycle, which provides the ATP and TCA needed for viral replication [52].

### Tick-borne encephalitis virus

Tick-borne encephalitis virus (TBEV) is a flavivirus transmitted by ticks. In rare cases, it can also be contracted after consuming unpasteurized milk from infected goats, sheep, or cows. TBEV causes tick-borne encephalitis (TBE), which is an infection of the central nervous system found in Europe and several regions of Asia. Although the disease can be prevented by vaccination, the incidence of the disease has been increasing significantly over the past few decades and is consequently a growing health problem in European and Asian countries. Moreover, as a result of increased human movement, TBE is also becoming a problem in other regions of the world (WHO: https://www.who.int/health-topics/tick-borne-encephalitis#tab=tab_1).

Most infections caused by TBEV are asymptomatic, whereas if the disease does occur, the incubation period of TBE usually lasts 7–14 days, followed by typical cold symptoms with fever and malaise over the next 1–8 days. However, in about 15% of patients, TBEV reaches the central nervous system, resulting in meningitis, encephalitis, myelitis, or radiculitis (WHO: https://www.who.int/health-topics/tick-borne-encephalitis#tab=tab_2). Even with treatment, many patients suffer from lingering symptoms, such as ataxia, headaches, and impaired concentration. ([141], WHO: https://www.who.int/health-topics/tick-borne-encephalitis#tab=tab_2).

After a bite form a TBEV-infected tick, the virus replicates first in the cells of the dermis and then in Langerhans cells, macrophages, and neutrophils [142]. Recognition of the virus by the innate immune system leads to migration of dendritic cells (DCs) to the primary site of infection. Once infected, these cells become activated and carry the virus through the lymphatic system to the regional lymph node and then to other organs. The introduction of TBEV into the body with milk, on the other hand, leads to viral replication in intestinal epithelial cells and subsequent infection of dendritic cells [143]. In the case of insufficient titers of antibodies that specifically neutralize TBEV, CNS infection occurs. How TBEV enters the brain is not fully understood, but it has been suggested that it can do so without destroying the BBB. Studies on primary human brain microvascular endothelial cells (HBMECs) have shown that TBEV-infected cells produce high viral titers and promote TBEV entry into the brain without disrupting the integrity of the BBB [144]. Instead, studies using mice have shown that an increase in BBB permeability occurs in the later stages of infection, accompanied by severe clinical symptoms and high virus titers in the brain. This is thought to be a consequence of overproduction of cytokines (TNF-α, IL-6, IFN-γ,) in the brain [145]. In the CNS, on the other hand, the virus localizes to neurons, which contributes to the inflammatory process manifested by cell dysfunction or degradation through lysis or necrosis/apoptosis [146]. Neuronal infection leads to the migration of T lymphocytes into the CNS, with a response of cytotoxic T lymphocytes necessary to remove the virus. In some cases, however, it can also lead to increased immunopathogenesis and neuronal damage [97]. Host cells have several defense strategies against viral infection. TBEV induces an innate and adaptive immune response at the site of infection; however, the virus has developed various strategies to block the host’s innate immune response, allowing the virus to replicate effectively in the originally infected cells. These actions include the NS5 protein, which is thought to be an antagonist of type I interferon (IFN). By inhibiting the JAK/STAT signaling pathway, it makes infected cells resistant to type I IFN [147]. However, pro-inflammatory cytokines, such as TNF-α, IFNα, IL-1β, IL-6, and IL-8 [148], and chemokines, such as CXCL10, CXCL11, CXCL12, and CXCL13 [149], are also detected in the cerebrospinal fluid of TBEV-infected individuals. IL-10 is elevated in the initial phase of TBE, but in severe course of disease, especially at later days of infection, IL-10 levels in the cerebrospinal fluid of TBEV-infected patients are reduced, which may contribute to a decrease in IFN-γ synthesis and act as an immunosuppression stimulant promoting inhibition of type 1 pro-inflammatory cytokine cascade. In addition, low levels of IL-10 may result in an increase in the activity of pro-inflammatory mediators, such as TNF-α, IL-6, and IFN-γ, which may favor a more severe course of TBE and promote decreased production of anti-TBEV antibodies [150].

**Fig. 6:**
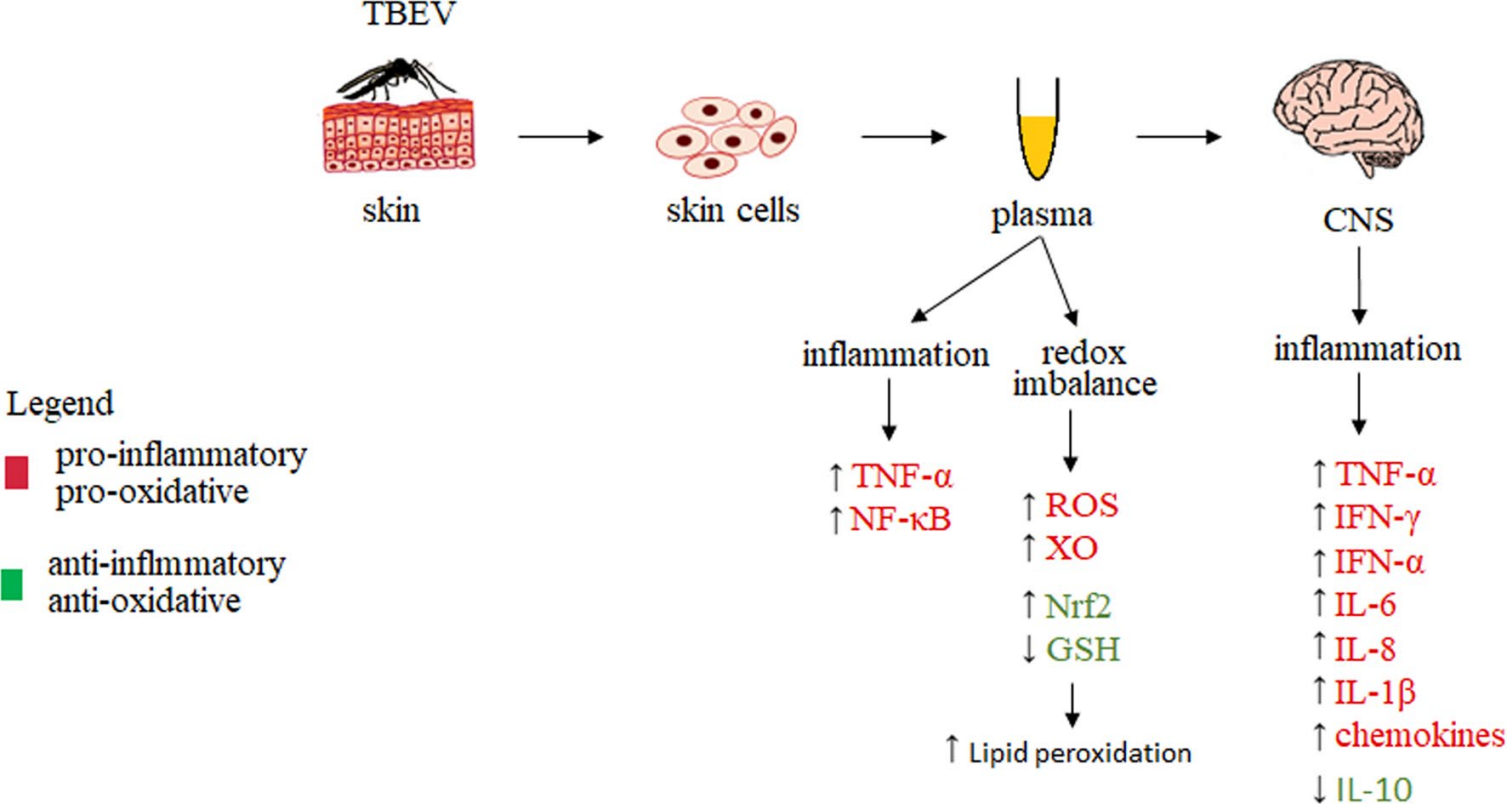
Metabolic changes occurring in the blood and central nervous system during Tick-borne encephalitis virus (TBEV) infection

Increased pro-inflammatory response in TBEV-infected patients is usually accompanied by overproduction of ROS, which should be compensated by the activation of one of the most important antioxidant mechanisms, i.e., the Nrf2/ARE pathway [151]. However, during TBEV infection, the activity of one of the primary pro-oxidant enzymes, i.e., xanthine oxidase, is also elevated, resulting in an increase in ROS levels [45]. Consequently, as a result of the pro-oxidative effect of ROS on protein structures, there is a decrease in the activity of enzymes related to the glutathione system and responsible for protecting, among others, phospholipids, such as glutathione peroxidase (GPx), reductase (GSSGR), and glutathione (GSH), resulting in reduced protection of brain lipid structures from oxidative damage [45, 100]. This results in, among others, oxidative modifications of polyunsaturated fatty acids (PUFAs), including both phospholipid and free PUFAs, leading to an increase in lipid peroxidation products caused by both oxidative fragmentation (4-HNE) and oxidative cyclization (8-isoPGF2α) in TBEV-infected individuals [45]. These changes further exacerbate oxidative stress, resulting in changes in the enzymatic metabolism of phospholipids with generation of lipid mediators including endocannabinoids and eicosanoids, which directly and indirectly, through the activation of membrane receptors mainly associated with G protein, enhance pro-inflammatory response with increased expression of transcription factor NF-κB and pro-inflammatory cytokine TNF-α (Fig. 6) [45].

### Zika virus

Zika virus (ZIKV) is a flavivirus transmitted by mosquitoes of the genus Aedes. However, perinatal transmission through sexual contact and the presence of ZIKV in milk have also been observed [152, 154]. Initial ZIKA virus infections were characterized by a mild course. However, the first outbreak due to Zika virus infection occurred on Yap Island (Federated States of Micronesia) in 2007, while another outbreak was reported in 2013–2014 in French Polynesia (WHO: https://www.who.int/newsroom/factsheets/detail/zikavirus?gclid=CjwKCAjwzuqgBhAcEiwAdj5dRtsigH_XKpDEufCGTodNW1RRasHGD__iQvBhzJbbd2GJ0vlreZF8xoCZ5wQAvD_BwE). In addition, in 2015 the WHO received the first reports of localized infection in Brazil, with the first instances of microcephaly in infants of mothers exposed to ZIKV during pregnancy reported in October. The rapid spread of infection in the Americas led the WHO to declare in February 2016 that Zika virus infection associated with neonatal microcephaly and other neurological disorders is a public health emergency of international concern [155]. It is now known that the clinical manifestations of classic Zika disease are characterized by fever, rash, conjunctivitis, arthralgia, and headache (CDC: http://www.cdc.gov/zika/symptoms/index.html) and may be accompanied by Guillain–Barré syndrome, acute myelitis, encephalomyelitis, encephalitis, meningitis and encephalitis, and sensory polyneuropathy [156, 157].

After a bite from an infected mosquito, Zika virus, similar to other flaviviruses, infects dendritic cells, where it then replicates and spreads through the blood to other organs [158]. ZIKV first binds to flavivirus-specific cellular receptors, which include DC-SIGN and phosphatidylserine receptor proteins, i.e., TYRO 3, AXL, TIM, and TAM [159]. These receptors facilitate ZIKV entry into macrophages, monocytes, neuronal progenitor cells (NPCs), and fetal cells, causing adhesion, migration, replication, and immune evasion, as well as cytokine release [159]. In skin fibroblasts, ZIKV induces the expression of pattern recognition receptors (PRRs), such as TLR3, RIG-1, and MDA5, enhancing the antiviral response against ZIKV infection [159]. However, studies using human embryonic kidney cells have shown that NS2A and NS4A proteins suppress NF-κB promoter activity by inhibiting signaling factors involved in the MDA5/RIG-I signaling pathway [160]. An early response to infection is the antiviral effect of type I interferon (IFN) produced by mammalian cells [161]. Zika virus’s non-structural proteins such NS1 and NS4B can inhibit type I IFN production, while NS2B and NS3, i.e., NS2B–NS3, inhibit JAK–STAT signaling by promoting Jak1 degradation [162]. IFN I transduces signaling through Janus kinases (Jak1 and Tyk2) and transcription signal transducers (STAT1 and STAT2), leading to interferon-stimulated gene (ISG) induction, which establishes an antiviral state of the cells, with inhibition of JAK–STAT signaling promoting viral replication [162]. ZIKV infection of human monocytes has also been shown to activate TLR2 signaling, leading to NF-κB activation and a strong NF-κB-dependent pro-inflammatory response, with increased production of TNFα, IL-1β, IL-6 and IL-10 in ZIKV-infected monocytes, which may be involved in the control of ZIKV proliferation. This is also accompanied by increased expression of STAT-dependent cytokines and CC chemokines, including IL-7, IL-15, CCL2, CCL3, CCl5, and CCL7 in ZIKV-infected monocytes [163]. Studies in ZIKV-infected mice have also shown high levels of TNF-α, IL-6, and IL-1β in the animals’ microglia [164]. Moreover, ZIKV infection induces an antiviral response to control virus replication in an IFN-independent manner, by inducing IL-27 expression [163].

**Fig. 7:**
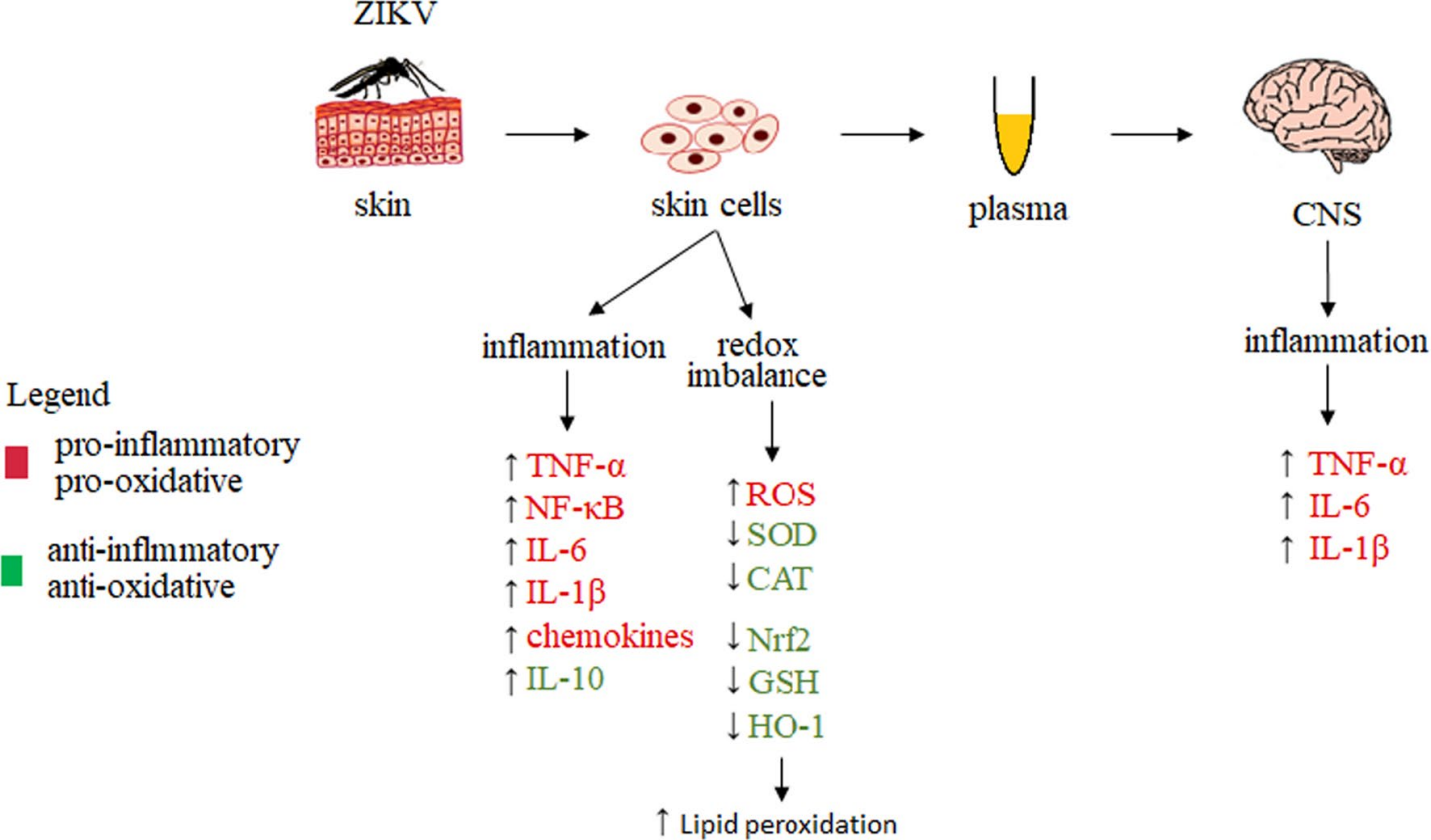
Metabolic changes occurring in vivo and in vitro in cells and the central nervous system during Zika virus (ZIKV) infection

ZIKV infection leads to an increase in ROS production by the body to combat the infection, with a concomitant inhibition of the activation of antioxidant transcription factor Nrf2 and the accompanying down-regulation of antioxidant protein gene expression and their biosynthesis (HO-1 SOD and CAT) [44], as well as a reduction in GSH levels [165]. Reduction in the level and activity of heme oxygenase-1, due to its participation in the reduction of ZIKV replication, promotes successful infection of the host by ZIKV [166]. Increased ROS generation with reduced antioxidant capacity leads to oxidative stress with enhanced lipid peroxidation and increased levels of its biomarker, i.e., MDA [44]. On the other hand, the reduced efficiency of transcription factor Nrf2 in ZIKV infection, which usually corresponds to an increased efficiency of pro-inflammatory transcription factor NF-κB, is evident in the increased generation of pro-inflammatory cytokines, such as TNF-α and IL-6, IL-1β, which recruit other cell types to the infected tissue and, by activating them, exacerbate inflammation. In addition, these mediators can also contribute to the activation of cell death pathways and further induction of oxidative stress, which promotes oxidative modifications of lipids and proteins, resulting in additional metabolic disorders in the patient’s body (Fig. 7) [44, 167].

In addition, in nerve cells, ZIKV increases the expression of cell death proteins, including: ZBP1, RIPK 1 and 3. Virus-infected nerve cells express the immune response gene 1 (IRG1), the product of which produces itaconate from cis-aconite, a component of the TCA cycle, which inhibits succinate dehydrogenase (SDH), thereby maintaining adequate levels of succinate, which keeps the nerve cells alive. Inhibition of SDH activity simultaneously inhibits ZIKV replication [168].

### Japanese encephalitis virus

Japanese encephalitis virus (JEV) is a flavivirus transmitted by Culex mosquitoes that causes Japanese encephalitis (JE) among people in Asian countries with an estimated 68,000 clinical cases each year, including 14,000–20,000 deaths. At the same time, 30–50% of those who survive the disease have permanent neurological or psychiatric sequelae (WHO: https://www.who.int/news-room/fact-sheets/detail/japanese-encephalitis).

JEV is a highly neuroinvasive pathogen with symptoms ranging from mild fever to aseptic meningitis or encephalitis, as manifested by altered sensation, seizures, and focal neurological deficits with acute flaccid paralysis that can result from anterior horn cell involvement, with 20–60% of patients showing a variety of movement disorders, especially transient features of parkinsonism and dystonia [169].

JEV introduced into the human body has been found to replicate first in dendritic cells and macrophages; it is then transmitted to local lymph nodes [170] and from there, with the help of newly generated virions or migrating infected immune cells—including dendritic cells and T cells—the virus spreads to the brain [171].

Studies in mice have shown that JEV induces inflammation that disrupts the integrity of the BBB and consequently increases brain levels of inflammatory mediators belonging to the Th1 immune response, as well as chemokines and cytokines, including CXCL10, CCL2, CCL3, CCL4, CCL5, TNF-α, IL-6, and IFN-γ in the CNS. Immediately after infection, there is an upregulation of C–X–C motif chemokine ligand 10 (CXCL10) and IFN-γ, which induces CXCL10 expression, with the highest amounts of inflammatory mediators observed just before BBB disruption [172]. In contrast, JEV-activated microglia release TNF-α and IL-1 β, which can provide protection against central nervous system infection, but can cause neuronal death [173]. In addition, mouse studies have shown that JEV infection induces expression of TLR3 and RIG-I receptors in the microglia of infected animals, which provide the first line of defense in the antiviral immune response through the release of cytokines and chemokines. In addition, it has been found that interaction of viral RNA with TLR3 and RIG-I leads to the activation of nuclear factor NF-κB and induction of expression of pro-inflammatory cytokines and chemokines, such as TNF-α, IL-6, and chemokine ligand 2 (CCL2) [174]. It has also been shown in in vitro studies that following viral infection of human or porcine dendritic cells, an increase in TNF and IFN-β production can be observed [175] (Fig. 7).

In addition, JEV has been shown to induce an inflammatory response that promotes increased levels of ROS in animal microglia [176], leading to increased activity of the primary antioxidant, SOD, in brain glial cells. The exact mechanism of SOD induction is not known, but it has been suggested that it may constitute a response to a strong inflammatory mediator or may be a compensatory mechanism to reduce the levels of superoxide anion radical, increased amounts of which have been detected in JEV-infected cells [177]. In addition, JEV infection decreases thioredoxin expression in human promonocyte cells [178], while JEV infection in rats also promotes a decrease in CAT, GPx, and GSH activity in the brain, which disrupts the homeostatic redox balance during infection and exacerbates oxidative conditions that promote lipid peroxidation and a consequent increase in MDA levels [101].

**Fig. 8:**
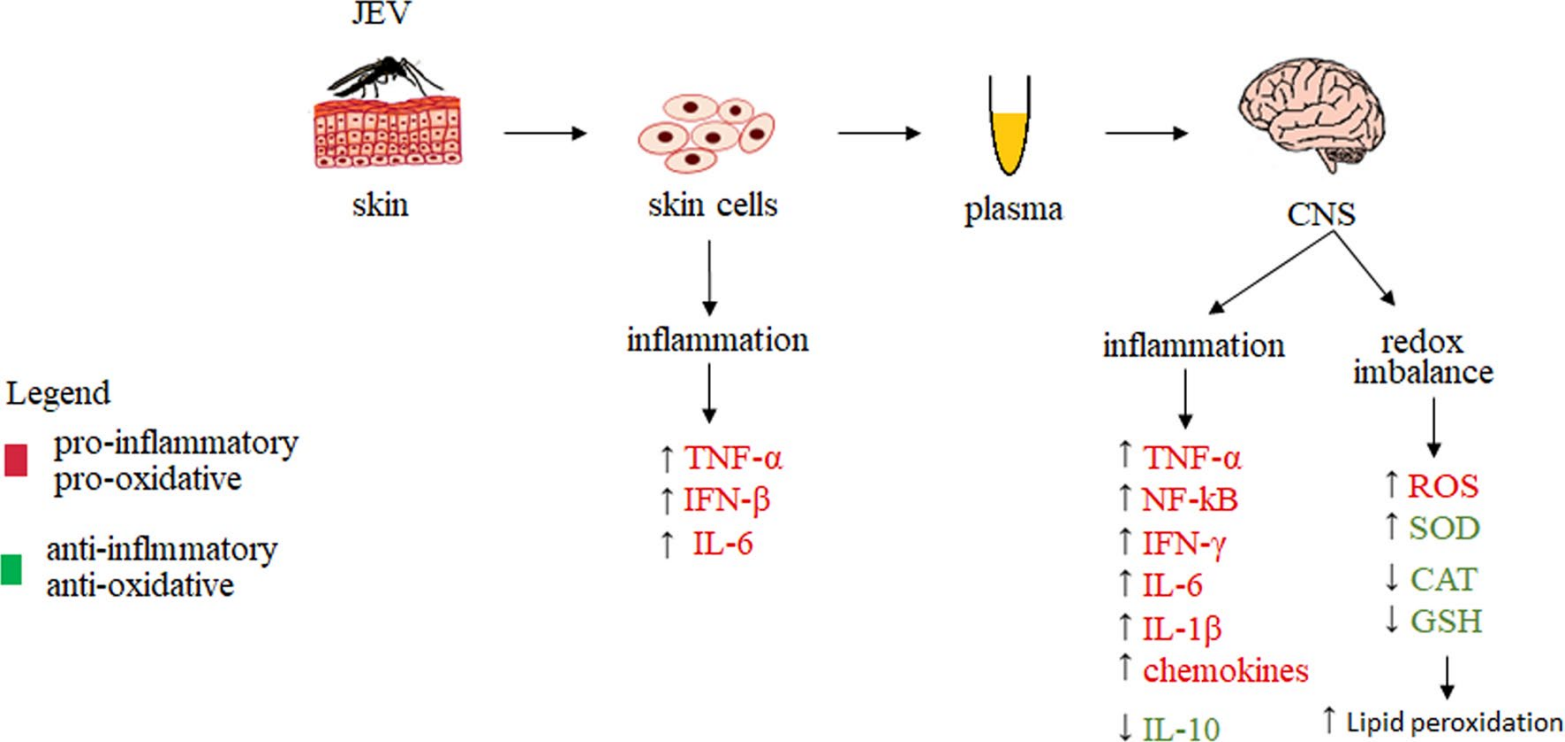
Metabolic changes occurring in vivo and in vitro in cells and the central nervous system during Japanese Encephalitis Virus (JEV) infection

JEV infection has also been shown to be associated with microglia activation in animals, resulting in increased levels of various pro-inflammatory mediators, such as inducible nitric oxide synthase (iNOS), cyclooxygenase 2 (Cox-2), interleukin-6 (IL-6), IL-1b, tumor necrosis factor alpha (TNF-α), and monocyte chemoattractant protein 1 (MCP-1) [176]. During JEV infection, decreased levels of anti-inflammatory cytokine IL-10 in the CNS are observed, which promotes the survival of neurons and all glial cells in the brain by blocking the action of pro-inflammatory cytokines and promoting the expression of cell survival signals [179]. In addition, in vitro studies on JEV-infected human neuroblastoma cells have shown impaired β-oxidation of long-chain fatty acids (PUFAs) and increased expression of interleukin 6 (IL-6) and tumor necrosis factor α (TNF-α). JEV non-structural protein 5 (NS5) was also found to interact with α and β subunits of hydroxyacyl-CoA dehydrogenase, two components of the mitochondrial trifunctional protein (MTP) involved in β-oxidation, and interfere with the catabolism of PUFAs [180]. It is thought that accumulated PUFAs may trigger oxidative stress in the CNS as well as activation of NF-κB and, consequently, increased production of pro-inflammatory cytokines, which contribute to JEV pathogenesis. It has been further suggested that this facilitates membrane proliferation and rearrangement in JEV-infected cells and likely contributes to both structural and metabolic damage to brain cells associated with Japanese encephalitis [180]. Moreover, in vitro studies using hamster kidney cells have shown that NS5 protein can suppress IFN-β expression by inhibiting IFN regulatory factor 3 (IRF3) and NF-κB (Fig. 8) [181].

Consequently, in JEV infection, although the initiation of immune responses by cells is an important protective mechanism of the CNS, the intensification of pro-inflammatory processes and, consequently, redox imbalance promote neuronal death, which can result in irreversible brain damage (Table 2).

**Table 2:** Metabolic changes flaviviruses infection occurring in vivo and in vitro in skin and blood as well as the central nervous system during infection

	WNV	DENV	TBEV	ZIKV	JEV
Pro-oxidants regulation					
Skin	**↑** ROS	**↑** ROS	No data so far	**↑** ROS, MDA	No data so far
Blood	N/A	**↑** ROS, MDA	**↑** XO, ROS, 4HNE, 8-isoPGF2α	N/A	N/A
OUN	N/A	**↑** ROS	N/A	N/A	**↑** ROS, MDA
Antioxidants regulation					
Skin	**↑** GSH ↓ Nrf2	**↓** Nrf2	N/A	**↓**SOD, CAT, Nrf2, GSH, HO-1	N/A
Blood	N/A	↓ GSH, SOD, CAT	**↑** Nrf2 **↓** GSH	N/A	N/A
OUN	N/A	N/A	N/A	N/A	**↑** SOD **↓**CAT, GSH
Inflammation					
Skin	**↑**TNF-α, NFκB, IL-6, IFN-β **↓**IL-10	**↑** TNF-α, IL-6, IL-1β, IL-10 chemokines, **↓** IFN-γ, IL-12	N/A	**↑** TNF-α, NF-κB, IL-6, IL-1β, chemokines **↓** IL-10	**↑** TNF-α, IFN-β, IL-6
Blood	N/A	**↑** TNF-α, NF-κB,IL-6	**↑** TNF-α, NF-κB	N/A	N/A
OUN	↑TNF-α,IL-6, chemokines	↑ IL-6, IL-8, CCL5	↑TNF-α, IFN-γ, IFN-α, IL-6, IL-8, IL-1β, chemokines ↓IL-10	↑TNF-α, IL-6, IL-1β	**↑** TNF-α, NF-κB, IFN-γ,IL-6,IL-1β, chemokines **↓** IL-10
Medical consequences of disease					
Skin	Rash without pruritus shit on the trunk and lower limbs	“white islands in a sea of red”	N/A	Itchy maculopapular rash	N/A
Blood	Thrombocytopenia	- **↑** Hematocrit - Thrombocytopenia - Leukopenia - **↑** ALT/AST	N/A	- Thrombocytopaenia - Leukopenia - Lymphocytosis - Monocytosis - ↑ALT/AST/LDH	- Thrombocytopenia - Leukopenia - Hyponatremia - **↑** ALT/AST
OUN	- Meningitis, - Encephalitis and/or myelitis	Encephalitis	- Meningitis, - Encephalitis - Cerebellitis - Myelitis	- Microcephaly with underdevelopment of the cerebrum - Psychomotor disorders - Spastic hemiplegia	- Encephalitis with disturbance of consciousness - Generalized seizures - Aseptic meningitis - Acute psychosis

## Final remarks

Flavivirus infections lead to inflammation and oxidative stress, both within the CNS and throughout the body, which affects both the cellular metabolism in the human body and the viral replication cycle. Limiting, as a result of pharmacotherapy, oxidative stress, including the exposure of the virus to ROS, may promote (via NF-κB) the multiplication of the pathogen, while oxidative stress may cause the death of the pathogen. In addition, overexpression of NF-κB leads to increased synthesis of cytokines that can block viral entry into cells. At the same time, oxidative stress in the host organism may exacerbate metabolic effects both in the CNS and in the whole organism, as indicated by the correlation of usually more severe infection with higher levels of ROS. This makes the multidirectional analysis of metabolic changes in the patient's body very important both from the point of view of diagnostics and personalized therapy, which should help in controlling the infection, including preventing adverse metabolic effects. It should also be taken into account that, apart from factors influencing redox balance and inflammation, non-structural proteins of flaviviruses, which help them to evade host defense mechanisms, seem to be an important therapeutic target. Therefore, a thorough understanding of the role of factors involved in the development of oxidative stress and inflammation during infection caused by flaviviruses and the determination of ways to consciously modify them may lead to the development of new diagnostic and therapeutic strategies in clinical management.

## Data Availability

Data are available in Corresponding Author.

## Abbreviations

4-HNE: 4-Hydroxynonenal
4-OI: 4-Octyl-itaconate
ADE: Antibody-dependent enhancement
ARE: Antioxidant response element
BBB: Blood–brain barrier
BDV: Borna disease virus
BMVECs: Brain microvascular endothelial cells
CAT: Catalase
CCL: CC-chemokine ligand
CDC: Centers for Disease Control and Prevention
CMV: Cytomegalovirus
CNS: Central nervous system
COX-2: Cyclooxygenase-2
CXCL: C–X–C motif chemokine ligand
DCs: Dendritic cells
DENV: Dengue virus
DMF: Dimethyl fumarate
EBV: Epstein–Barr virus
ECs: Endothelial cells
GLUT1: Glucose transporter 1
GPX: Glutathione peroxidase
GSSGR: Glutathione reductase
GSH: Glutathione
HAV: Hepatovirus A
HBMEC: Human brain microvascular endothelial cells
HHV6: Human herpesvirus type 6
HIV: Human immunodeficiency virus
HK2: Hexokinase 2
HSV: Herpes simplex virus
IL: Interleukin
iNOS: Inducible nitric oxide synthase
IFN-γ: Interferon-gamma
IPS-1: Interferon-beta promoter stimulator 1
IRF: Interferon regulatory factor
ISG: Interferon-stimulated gene
JEV: Japanese encephalitis virus
LAMs: Leukocyte adhesion molecules
LANA: Lysine acetylation of the nuclear antigen
LCMV: Lymphocytic choriomeningitis virus
LDH: Lactate dehydrogenase
MAV-1: Mouse adenovirus 1
MCP-1: Monocyte chemoattractant protein 1
MDA: Malondialdehyde
MDA5: Melanoma differentiation-associated gene 5
MeV: Measles virus
MMF: Monomethyl fumarate
MTP: Mitochondrial trifunctional protein
MuV: Mumps virus
NF-κB: Nuclear factor kappa B
NPCs: Neuronal progenitor cells
Nrf2: Nuclear factor erythroid 2-related factor 2
NMJs: Neuromuscular junctions
NO: Nitric oxide
NS: Non-structural protein
ORF: Open reading frame
PAMPs: Pathogen-associated molecular patterns
PNS: Peripheral nervous system
PRRs: Pattern recognition receptors
PUFAs: Polyunsaturated fatty acids
RIG-I: Retinoic acid-inducible gene I
RIPK3: Receptor-interacting protein kinase-3
RLRs: RIG-I-like receptors
RNS: Reactive nitrogen species
ROS: Reactive oxygen species
sMaf: Maf proteins
SDH: Succinate dehydrogenase
SOD: Superoxide dismutase
STAT-1: Signal transducer and activator of transcription 1
SSPE: Subacute sclerosing panencephalitis
TBE: Tick-borne encephalitis
TBEV: Tick-borne encephalitis virus
TCA: Tricarboxylic acid
TJs: Tight junctions
TLRs: Toll-like receptors
TNF-α: Tumor necrosis factor alpha
UTRs: Untranslated regions
VSV: Vesicular stomatitis virus
VZV: Varicella-zoster virus
WNV: West Nile virus
ZIKV: Zika virus
